# Additively Manufactured Parts Made of a Polymer Material Used for the Experimental Verification of a Component of a High-Speed Machine with an Optimised Geometry—Preliminary Research

**DOI:** 10.3390/polym13010137

**Published:** 2020-12-31

**Authors:** Artur Andrearczyk, Bartlomiej Konieczny, Jerzy Sokołowski

**Affiliations:** 1 Institute of Fluid Flow Machinery, Polish Academy of Sciences, 80-231 Gdansk, Poland; 2 University Laboratory of Material Research, Medical University of Lodz, 92-213 Lodz, Poland; bartlomiej.konieczny@umed.lodz.pl; 3 Department of General Dentistry, Medical University of Lodz, 92-213 Lodz, Poland; jerzy.sokolowski@umed.lodz.pl

**Keywords:** additive manufacturing, polymer resin, material jetting, turbomachinery, optimization

## Abstract

This paper describes a novel method for the experimental validation of numerically optimised turbomachinery components. In the field of additive manufacturing, numerical models still need to be improved, especially with the experimental data. The paper presents the operational characteristics of a compressor wheel, measured during experimental research. The validation process included conducting a computational flow analysis and experimental tests of two compressor wheels: The aluminium wheel and the 3D printed wheel (made of a polymer material). The chosen manufacturing technology and the results obtained made it possible to determine the speed range in which the operation of the tested machine is stable. In addition, dynamic destructive tests were performed on the polymer disc and their results were compared with the results of the strength analysis. The tests were carried out at high rotational speeds (up to 120,000 rpm). The results of the research described above have proven the utility of this technology in the research and development of high-speed turbomachines operating at speeds up to 90,000 rpm. The research results obtained show that the technology used is suitable for multi-variant optimization of the tested machine part. This work has also contributed to the further development of numerical models.

## 1. Introduction

Additive manufacturing (AM) technologies are increasingly used in scientific research. They also offer a practically unlimited number of application possibilities for the industry. Several key emerging trends can be identified, such as the combination of manufacturing techniques and processes, the increasing modularity of machines, mass production and the adaptation of AM techniques for use in selected industrial branches. Currently, AM is most often used in the medical industry [1,2,3], and its rapid growth is taking place during the COVID-19 [4] pandemic to produce all possible elements used to combat the pandemic and support the production of life-saving components [5]. A wide range of applications can be observed in industries such as stomatology [6,7], power engineering [8], aerospace industry [9] and even the automotive industry [10,11]. AM methods used to manufacture real 3D objects using the layer-by-layer technique use a few types of building materials, namely metals, polymers, ceramics and glass [12,13]. Added missing references Although metal AM technologies offer relatively high manufacturing accuracy, they are rarely used to produce precision parts because surface treatment is required to reduce roughness. AM technologies that use polymers make it possible to print parts with high accuracy without the need for further processing. Unlike AM technologies capable of making parts from metals powders, among which Laser Powder Bed Fusion (LPBF) [14] is currently the most widely used and offer a similar level of manufacturing accuracy, polymer-based AM technologies use a wide range of printing methods such as Material Extrusion(ME), Binder Jetting (BJ), Material Jetting (MJ), Vat Photopolymerization (VP) and hybrid technologies, with different levels of manufacturing accuracy [15]. Some polymer-based technologies, despite their weaker mechanical properties than metal-based 3D printing technologies, successfully find application in the prototyping of machine parts [16,17].

There are three types of polymers used in AM machines, namely thermoplastic [18], thermohardening and light-curing polymers [19,20]. The evaluation of their properties makes it possible to choose the most suitable building material, depending on the required characteristics of the element to be manufactured. The mechanical and chemical properties of the materials make them suitable for industrial use. Thermoplastics are best suited for the production of finished products and testing prototypes. They have good mechanical properties, high impact strength as well as high abrasion and chemical resistance. They can also contain coal, glass or other additives, which improve their physical properties [21]. The most commonly used thermoplastics in the industry are nylons—poliamids (PA) and elastomers—thermoplastic polyurethane (TPU) [22], which have similar mechanical and physical properties (especially ultimate tensile strength which is very important in the design process of impellers) to materials used for 3D printing using ME technologies such as polylactic acid (PLA), acrylonitrile butadiene styrene (ABS), polyethylene terephthalate glycol (PETG), polyetherimide (PEI)—an amorphous material better known by its trade name ULTEM, acrylonitrile styrene acrylate (ASA) [23,24], which are also used due to economic reasons and their shorter printing process. One of their disadvantages is low printing accuracy. Thermohardening plastics (resins) are better suited for applications where aesthetics and high manufacturing accuracy are important, as they can be used to produce parts with smooth surfaces, like during injection moulding [25]. They generally have high stiffness but are more brittle than thermoplastics, so not all of them are suitable for functional applications, even those mimicking the properties of ABS and polypropylene (PP) [26], which are used, for example, in the manufacture of dental prostheses and implants [27]. The last group are light-curing polymers, which currently offer one of the highest printing accuracies (without post-treatment) thanks to the minimum height of a single layer of 16 μm [28]. Depending on the manufacturer and building material used in this technology, it is possible to distinguish aesthetic and functional resins with strictly defined mechanical properties. The strongest of these resins are similar in strength to thermoplastics. In this study, this type of manufacturing technology has been used because of its advantages.

In order to increase the performance and efficiency of both prototypical and commercially available high-speed power generation machines [29], it is essential to optimise their main components in this respect [30]. In turbomachines, the fluid-flow system and the geometry of the blades are usually optimised [31]. Sometimes strength optimisation is also performed on components that must withstand harsh operating conditions to increase performance and achieve higher operating parameters [32]. During the process of optimising the geometrical parameters of the fluid-flow system, a number of optimisation methods are used, which can be divided into the following three groups: deterministic methods (for example, gradient methods, simplex methods and simple search methods) [33], stochastic methods (genetic algorithms and annealing) [34] and hybrid deterministic-stochastic methods [35]. The main approach in the optimisation of blade systems is based on the concept of target function optimisation [36], which represents a certain general property of the fluid-flow system, for example, enthalpy loss in the turbine stage(s). So far, the optimisation of components such as the rotor discs of high-speed turbomachines has been experimentally verified only to the extent that enables the creation of such a geometry using the available manufacturing technologies. The rest of the optimised surfaces were either very difficult or very expensive to produce, for example, blades with less than 1 mm clearance or blades with a very complex profile [37]. Such obstacles prevented any experimental verification, leaving the optimisation outcome in the form of theoretical considerations. The emergence and rapid development of 3D Printing technologies and devices have made it possible to take a closer look at some AM methods, in terms of their applications in this field, and to enhance the process of verifying and optimising the geometries in high-speed machines.

The aim of the paper is to present a tool for verifying the optimised shape of the blades of rotor discs (i.e., turbine disc and compressor disc), the manufacture of which is either very difficult or impossible using the conventional production methods (that is, machining, CNC milling, etc.). The results and considerations presented in the paper constitute a continuation of research on the use of polymer-based AM technologies for the manufacture of prototype parts of high-speed machines [11,38].

## 2. Materials and Methods

### 2.1. Manufacturing Technology

After a thorough examination of the available AM technologies, MJ technology was selected and used in our research. AM technology uses additive manufacturing to produce models. The building material, which is deposited on the build platform by a printhead, is automatically cured by a UV lamp. The material is precisely deposited layer-by-layer using printing jets. The device builds wax supports, which must be melted after the printing process. The MJ AM process is fully automated and the role of the machine operator is limited to placing models on a virtual platform and uploading them to an AM machine. A ProJet 3500 HD Max printer (3D Systems, Rock Hill, SC, USA) was used to manufacture rotors in this work.

**Figure 1:**
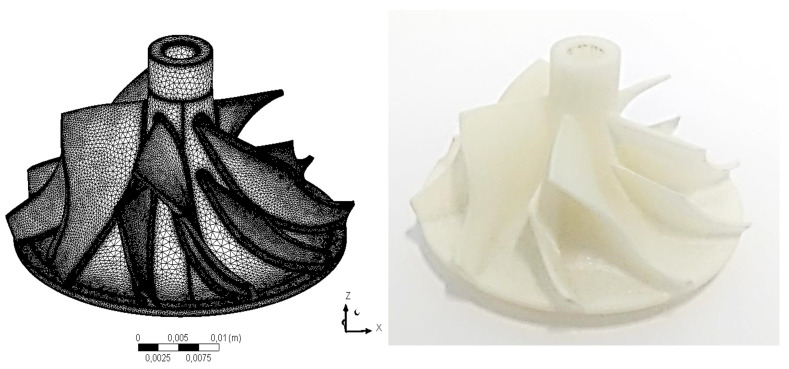
Model of compressor disc obtained by 3D scanning (**left**) and disc created using the MJ method (**right**).

MJ technology makes it possible to select several types of light-curing resins with different mechanical properties. Due to the specified operating conditions [11], the most resistant material with the catalogue name of VisiJet M3-X (3D Systems, Rock Hill, SC, USA) with a tensile strength of 54 MPa and a softening temperature of 88 ${}^{\circ }$C [39] was used in the research presented in this paper. The material used in the research was chosen from two of the most durable materials offered by the manufacturer of the selected printing technology. The materials were experimentally tested to determine their tensile strength and maximum operating temperature and the results are described in papers [11,39]. The aluminium compressor disc comes originally from an industrial turbocharger used in this research and no design data is available. The original compressor disc was scanned with a 3D laser scanner and subjected to a numerical strength analysis [40] to first determine its operating range. The disc model was then converted to the required .stl format using the Autodesk Inventor environment (Autodesk, Mill Valley, CA, USA) and the disc was created using the method described above. The disc was printed with the highest possible accuracy (XHD mode) using the mentioned printer, and the height of a single layer was 16 μm at a resolution of 750 DPI. The post-treatment involved melting the wax in an oven at a temperature of 60 ${}^{\circ }$C. The scan result and the 3D printed disc are shown in Figure 1.

After printing the rotor disc, the main dimensions of both discs (aluminium and polymer disc) were measured using precise micrometres and diameter measuring devices. The external dimensions did not differ by more than 0.01 mm. A surface quality test was also carried out using a surface roughness meter—MarSurf PS1 (MAHR/Unipre, Werl, Germany). Slight differences in the values of the surface roughness coefficient (R${}_{a}$) were observed, where for the aluminium disc its value was around 0.6 μm and for the polymer disc 0.5 μm. Such a small difference should not affect the operational performance of the discs.

### 2.2. Numerical Analysis

A numerical model was created using reverse engineering. After performing a 3D laser scan, a 3D point cloud was obtained. This cloud was used to create a 3D model of the machine (using the appropriate software). The model was imported into ANSYS. In the numerical model, the diameter of the compressor disc was the same as in reality (42.5 mm). In order to simulate the performance of the recreated geometry, a RANS (Reynolds-Averaged Navier-Stokes) analysis was performed using the commercial software ANSYS CFX (ANSYS, Academic research 2014, Canonsburg, PA, USA) [41]. A second-order space discretization was applied. The working fluid (air) was modelled as an ideal gas. This was justified because the maximum pressure ratio was approximately 3. Steady-state simulations were conducted with direct interpolation between the rotor nodes and the volute domains. The Shear Stress Transport turbulence model with an automatic wall function was applied. The computational mesh for a single rotor channel was created in ANSYS Turbogrid [41], which generates hexahedral grids. The entire rotor domain was prepared by means of a circular pattern of the obtained mesh that was characterised by matching periodic nodes. The quality of the mesh was sufficient, as the minimum orthogonality angles were greater than 22${}^{\circ }$ and the maximum aspect ratios were lower than 1000 at the boundary layer. The mesh quality was calculated as the minimum ratio of the height to the base length of each side (normalised to 1) of an element. The mesh of the compressor volute consisted of tetrahedral elements of minimum quality equal to 0.05. The numerical model used for the flow analysis is described in detail in [11].

Due to the influence of the inlet air temperature of the compressor on the measurement results, a decision was made to carry out a flow analysis for two temperatures values, taking into account the minimum and maximum ambient temperatures prevailing in the laboratory during the tests. Temperature values of 15 ${}^{\circ }$C and 27 ${}^{\circ }$C were taken into account during the setting of the boundary conditions. The rotational speed range was selected on the basis of previous experiments [11,38]. In the speed range of 60,000–90,000 rpm, the machine with a polymer disc functioned properly compared to the turbocharger with the original aluminium disc.

### 2.3. Experimental Setup

**Figure 2:**
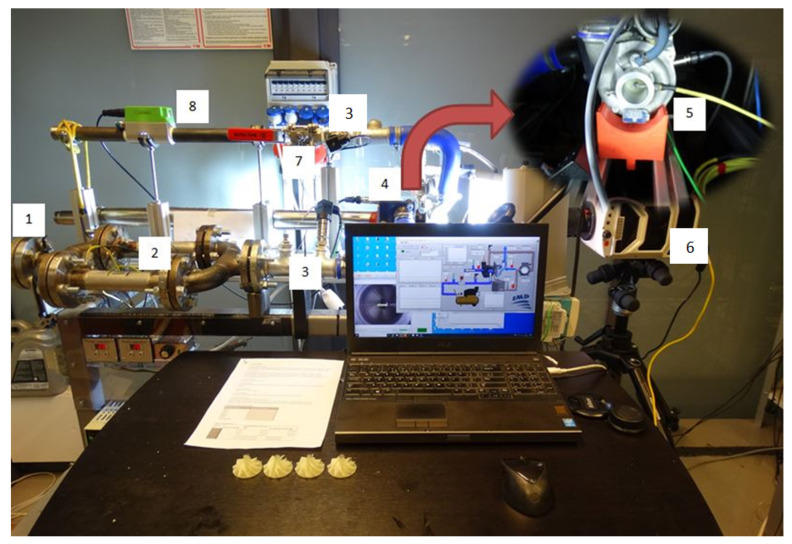
Test stand with a measurement system for registering and determining the flow characteristics of the turbocharger compressor. 1—air supply side with a throttle valve; 2—air pre-heating system; 3— temperature and pressure sensors; 4—turbocharger; 5—speed sensor; 6—high-speed camera; 7—compressor throttle valve; 8—flowmeter.

In order to determine the flow characteristics of the turbocharger compressor with a polymer disc, a test stand was used to supply the turbine with compressed air heated to a temperature of 50–150 ${}^{\circ }$C. The turbocharger used in this research was an industrial automotive turbocharger from Renault Clio II 1.5 dCi 82HP (Renault, Boulogne-Billancourt, France). It was not possible to use combustion gases to power the machine due to the mechanical properties of the polymer resin of which the rotor disc used in the research is made. Therefore, an in-house-designed air preheater (2) was used at the turbine inlet to avoid this situation. The air preheater consists of two heaters connected in parallel (each with a power of 3.3 kW) and is controlled by a voltage regulator. An oil-free compressor with a maximum operating pressure of 10 bar was used to supply the turbine. During the tests, the supply pressure was set to 8 bar. The inlet pressure of the turbine was controlled by an analogue throttle valve (1), which allows for precise control of the rotational speed of the turbine. Since the research presented in this paper was not intended to determine the power of the turbine, it was not necessary to measure its mass flow rate. The mass flow rate measurements were carried out only on the compression side. The test stand used for the research is shown in Figure 2.

An extremely important element of the turbocharger is the lubrication system, which, if not properly controlled, may lead to energy losses resulting from friction of the lubricating film and throttling the operation of the machine. The lack of or poor lubrication pressure of bearings used in the machine may lead to its damage. This was achieved by using an oil pump controlled by an inverter, which kept the oil pressure in the range of 2.5–4 bar (depending on the rotational speed). An electric heater was installed in the oil tank in order to maintain an appropriate temperature level of the lubricant through hysteresis (60–80 ${}^{\circ }$C).

In order to make it possible to measure several characteristics points, a throttle valve (7) was used at the compressor outlet and it was possible to change the opening level by 10% at a time. The experimental research assumed to analyse six characteristics points for each rotational speed (0—without throttling, 1–5—with throttling). Due to the lack of reaction of the system to throttling at a 10% valve closure level, throttling began at a 20% closure level and ended at a 60% closure level. The upper limit of the closure level of the valve was adopted for safety reasons. During previous experimental tests, above this level (70%), the turbocharger operated in the surge mode [42], which might have lead to damage to the machine if the operation had been longer.

During testing of this disc, the same temperature was set for the air supplied to the turbine as when testing the aluminium disc. In this way, similar operating temperatures of the machine were obtained in both tests. The measurements were carried out using a program created in the LabVIEW programming environment, coupled with the National Instruments measurement system. The following measuring transducers were used in the measurements: K-type thermocouples (3) with programmable transducers (TMD20) (CZAKI, Rybie, Poland), with an accuracy cold junction compensation ±1 ${}^{\circ }$C; laser rotational speed sensor (5) (Optel Thevon, Montreui, France) that enables measurement in the range up to 1 million rpm; EE741 (Introl, Katowice, Poland) thermal flow meter (8) that allows mass airflow measurement with an accuracy of 1%. Compression level was measured using NPX (Peltron, Wiazownia Koscielna, Poland) pressure transducers (3) with an accuracy of 0.5%.

The test stand, in accordance with the operating conditions, was also adapted to perform destructive tests. For this purpose, a Phantom v2511 (Vision Research, Wayne, NJ, USA) high-speed camera (6) was used to capture fast-changing phenomena (up to 1 million frames per second) and additionally the casing of the turbocharger (4) was equipped with vibration acceleration sensors (PCB Piezotronics, New York, NY, USA). Based on previous analyses and experimental tests [40], it had been determined that at a speed of 120,000 rpm, the disc is torn into pieces. Since experimental tests were carried out on a polymer disc to determine the operating characteristics of the compressor, a decision was made to re-verify the destructive tests (previous tests were performed on unused ten polymer discs).

## 3. Results

### 3.1. Numerical Results

**Figure 3:**
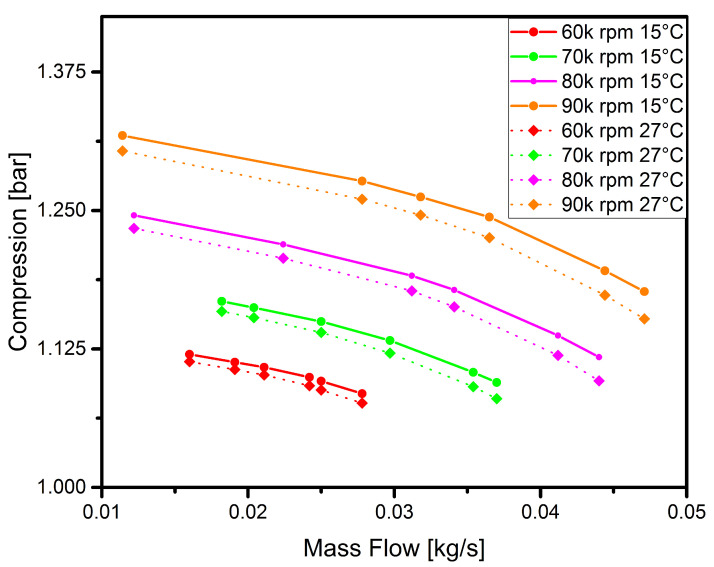
Numerically obtained flow characteristics of the compressor for two air temperatures measured at the inlet: 15 ${}^{\circ }$C (solid line) and 27 ${}^{\circ }$C (dotted line).

The results were determined for four rotational speeds that lie within the range of the correct operation of the polymer compressor disc. The results of the numerical flow analysis are shown in Figure 3.

It is clear from the analysis that the higher the temperature at the compressor inlet, the greater the compression. At a speed of 60,000 rpm, the effect of temperature is very small or even negligible. It can be noticed that the temperature increases linearly with the rotational speed. This is due to the greater temperature difference resulting from the increase in temperature at the compressor outlet (due to the compression of air). Given the imperfection of the numerical model of the disc in relation to the real disc, such results make it possible to verify whether at least some of the characteristic points fall within the range of the numerical analysis. The model [11] does not take into account details such as blade clearance and needs to be extended and improved, and this is being planned for study in future papers.

### 3.2. Flow Characteristics

**Figure 4:**
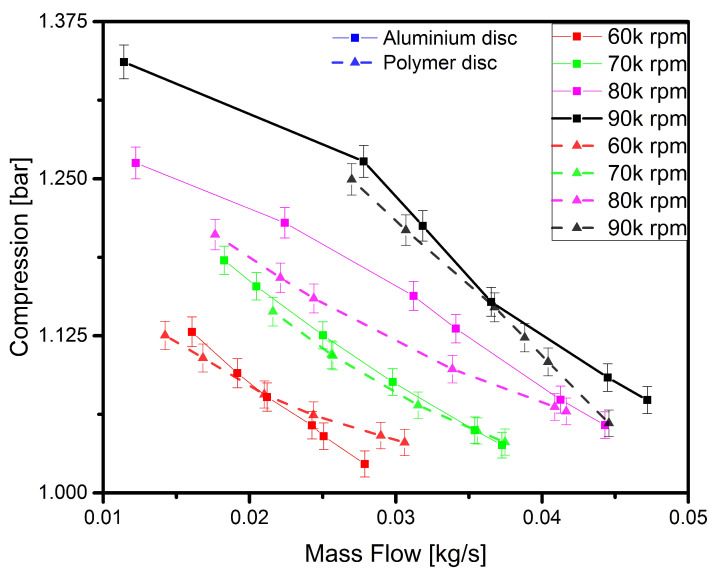
Compressor characteristics obtained experimentally with an aluminium (solid line) and polymer (dotted line) disc.

The experimental research was focused on analysing the performance characteristics of a turbocharger with an aluminium or polymer disc. Turbochargers are used in combustion engines to improve their operational performance by adding to them the pressure built up by compression (the so-called ‘charge pressure’). A compressor functions properly when the pressure at its outlet is increased while the pressure at the compressor outlet is throttled (i.e., mass flow is reduced). This measurement was carried out to enable the proper functioning of the polymer compressor disc to be verified by comparing its performance with that of a properly functioning aluminium disc. Each performance characteristics consisted of six measurement points. The first tests focused on the aluminium disc and its operation in the selected range of rotational speeds. Then, tests were carried out under the same operating conditions using the polymer disc. Each of the measurement points was recorded after the operating parameters became stable at a predefined rotational speed and throttling level. The stabilisation period varied but was always within a 60-second time interval. The results obtained vary considerably depending on the rotational speed and the throttling level at the compressor outlet. The results of the above-mentioned experiments are shown in Figure 4.

In the figure, the curves obtained from the the experiment carried out with a polymer and aluminium disc are similar for each rotational speed and the results are the most similar between the second and fourth points (throttling level). Based on previous research regarding the dynamic analysis of the turbocharger operating with the polymer disc, it was at these points that the machine’s operation was most stable in terms of vibration. However, in the 80,000–90,000 rpm speed range, a decrease in the performance of the compressor blades can be observed, which is reflected in lower compression levels recorded at the last two compressor outlet throttling levels. In previous research conducted on a polymer disc, similar results were obtained, which indicated the malfunction of the rotor disc blades resulting from the appearance of deformations at their tips.

**Figure 5:**
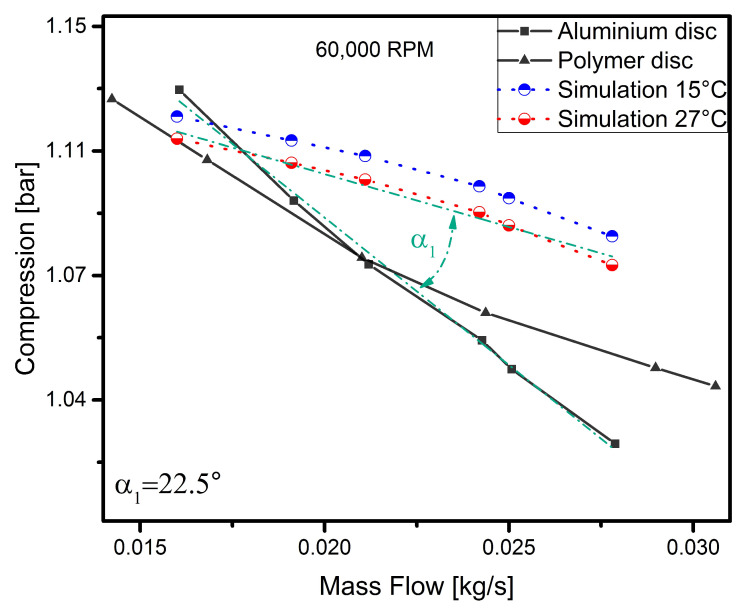
Simulation vs. experimental results for a speed of 60,000 rpm.

**Figure 6:**
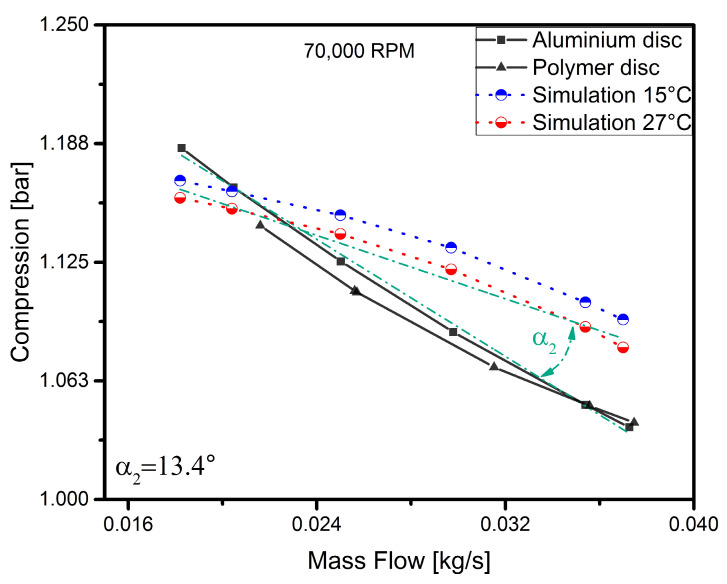
Simulation vs. experimental results for a speed of 70,000 rpm.

**Figure 7:**
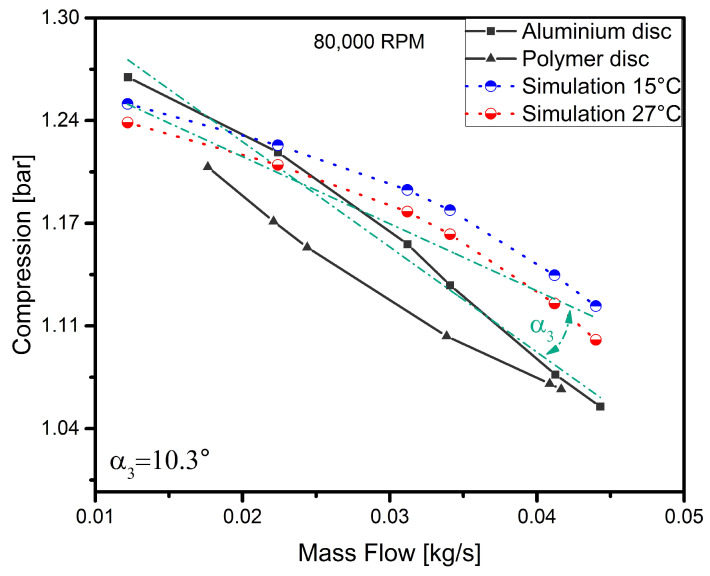
Simulation vs. experimental results for a speed of 80,000 rpm.

**Figure 8:**
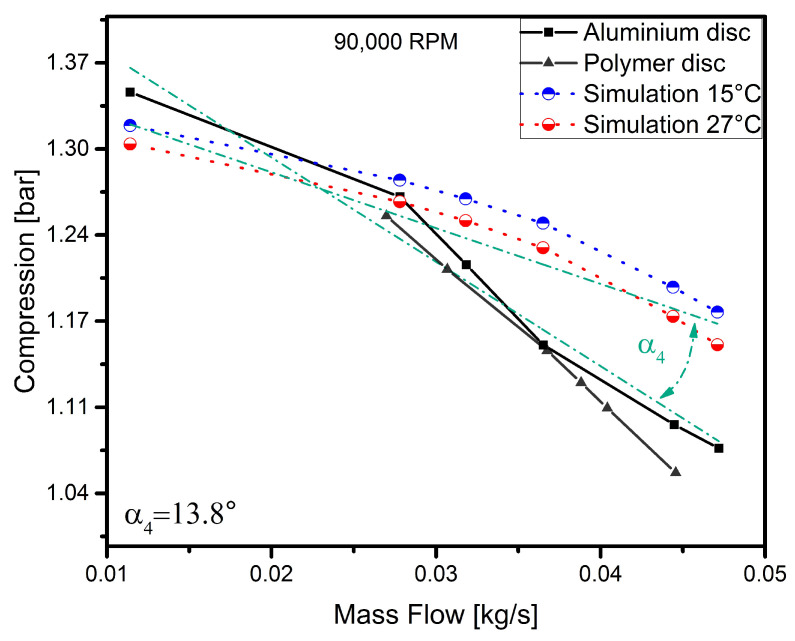
Simulation vs. experimental results for a speed of 90,000 rpm.

To discuss the results in more detail, each curve representing the operating characteristics of the disc for a given rotational speed has been analysed separately. In order to verify the model and the experiment, simulation curves determined earlier were added to the results obtained. The results are shown in Figure 5, Figure 6, Figure 7 and Figure 8.

The results presented in Figure 5 show the operating characteristics of the compressor with an aluminium and polymer disc recorded at a speed of 60,000 rpm. The nature of the experimental curves slightly differs from each other (the maximum difference between the measured values does not exceed 3%), which makes it possible to affirm that the machine with the polymer disc behaves in a similar way to the one with the aluminium disc under these operating conditions. At this speed, both discs achieve very similar charge pressure values. However, there is a clear difference between the results of the experiment and the simulation. At the last point (the last throttling level), the experimental curve intersects the simulation curves. Since numerical calculations were performed for the aluminium disc (due to the lack of complete data regarding the polymer disc model in the literature), the inclination angles of the curves obtained from the experimental results regarding the aluminium disc were compared to the determined range of curves obtained from numerical calculations. To do this, the linearity of the nature of the operation was assumed and the crossed lines (green dash-dotted lines) were determined based on a least-squares regression fit. In the case of numerically determined characteristics, the regression fit were assumed between the curves for temperatures of 15 ${}^{\circ }$C and 27 ${}^{\circ }$C. The angle of inclination between the curves (α) is 22.5${}^{\circ }$. It is probably due to the fact that the numerical model does not take blade clearance into account. Due to the dynamically stable operation of the machine, a decision was made to analyse the variations in the angle of inclination between the curves obtained for the particular rotational speeds.

At a speed of 70,000 rpm (Figure 6), it can be observed that the nature of the operation of both discs is very similar. Under these operating conditions, the results obtained for the polymer disc are to a large extent similar to those obtained for the aluminium disc. For these operating parameters, the operation of the machine is still stable. In this graph, there are more points where the simulation curves intersect the experimental curves. The angle between the lines decreased to 13.4${}^{\circ }$ as the rotational speed was increased.

The curve representing the operating characteristics of the machine with the polymer disc obtained for a speed of 80,000 rpm (Figure 7) has a concave shape—as in the previous cases, while the curve obtained for the aluminium disc has changed in nature and took a slightly convex shape. This shape demonstrates that the nature of the operation of the turbocharger compressor is typical [43]. Nevertheless, at this speed, the parameters achieved by both discs are similar. It can also be seen in this graph that the curves determined experimentally and by simulation, representing the nature of the operation of the machine, are broadly similar. Once again, there is a decrease to 10.3${}^{\circ }$ in the angle of inclination of the curves compared. This may indicate that this parameter depends on the speed of rotation. In addition, the experimental curve and the simulation curves are more similar than in the previous cases. For these operating parameters, there was also a slight increase in the level of vibration resulting from the bearing system used. From a dynamic point of view, the operation of the turbocharger was still stable and its vibration level was safe.

**Figure 9:**
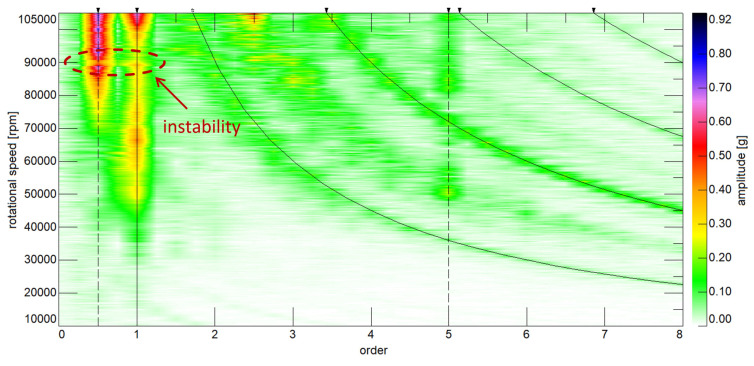
Dynamic characteristic of turbocharger with aluminium compressor disc presented in the form of a colourmap (speed range: from 10,000 rpm to 105,000 rpm).

The last curve, obtained for a speed of 90,000 rpm, is shown in Figure 8. As can be seen from the graph, the polymer disc only operates efficiently in the first half of the mass flow range and achieves a much lower charge pressure than the aluminium disc. Both experimental curves are slightly convex in shape, indicating that the nature of the operation of the two discs is similar. At this speed, the dynamic tests of the machine showed an increase in vibrations resulting from the operation of the bearings and their overlap with the radial vibrations stemming from the rotational speed (unbalance), which caused that the operation of the machine was unstable. The start of the unstable operation of the turbocharger is marked with a red circle in Figure 9. The figure also shows the dynamic results regarding the entire run-up of the turbocharger up to the rupture of the polymer rotor. But for this to have become possible, the vibration amplitude of the machine had to be presented as a function of the frequency components (order) for each rotational speed. A slight increase (to 13.8${}^{\circ }$) in the inclination angle of the simulation curves was also observed, which according to the authors, may indicate that the unstable operation of the machine affected this parameter. However, to confirm this, further testing is needed. Despite the increase in the angle of inclination, the nature of the simulation curves and the experimental curves is still broadly similar and results from not taking into account blade clearance in the numerical model.

### 3.3. Destructive Tests

**Figure 10:**
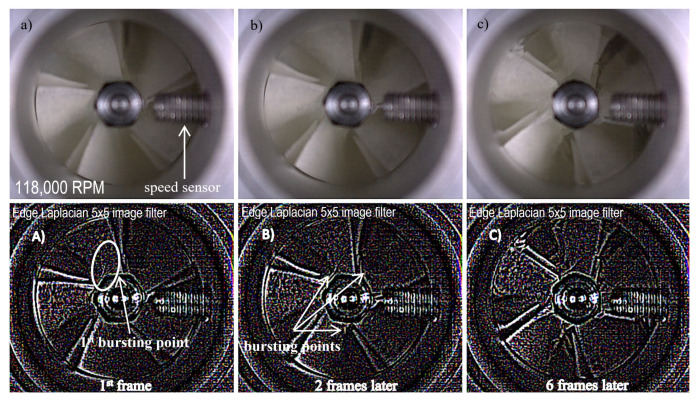
Images obtained at a speed of 118,000 rpm—original images recorded by a camera (top images: (**a**) 1^st^ frame, (**b**) 2 frames later, (**c**) 6 frames later) and images obtained after using the edge Laplacian filter (bottom images: (**A**) 1 ^st^ frame, (**B**) 2 frames later, (**C**) 6 frames later).

Paper [40] discusses a strength analysis performed on a polymer disc and dynamic tests in which an unused rotor was ruptured. A decision was made to repeat this research using the rotor on which the above-mentioned tests were carried out. In order to carry out destructive tests, the same numerical model and the same measuring apparatus were used as in the case of the first series of destructive tests described in paper. As dynamic testing was not the purpose of the studies described in this section, a decision was made to only present the rupture result and the data regarding the rotational speed at which the polymer disc was destroyed. In order to conduct a thorough verification, seven rotors were printed using a polymeric material and subjected to tests. As the results were very similar, it was decided to present only one case study. Figure 10 shows the frames recorded by an ultra-high-speed camera (Phantom v2511). The recording speed was 150,000 frames per second.

The value of the rotational speed at which the centrifugal force caused the permissible stresses to be exceeded (and the rupture of the disc) was also registered; under experimental conditions, this speed was between 115,000 and 120,000 rpm. The use of the edge Laplacian 5 × 5 image filter made it possible to highlight the first and other crack spots. As can be seen in Figure 10A, looking at the first bursting point, the disc broke in its central part, indicating that the maximum stresses stemming from the centrifugal force were exceeded at this point. The propagation of the crack is illustrated in Figure 10B,C. Compared to the results obtained previously, there was a slight change in the value of the speed at which the polymer disc broke. Subjecting the disc to other tests before subjecting it to destructive tests affected its strength.

## 4. Discussion

After analysing the obtained results, the polymer disc was found to function properly, as opposed to the aluminium disc. The polymer disc performed best in the speed range of 60,000 to 80,000 rpm. At a speed of 90,000 rpm, it had a lower performance and its operating characteristics were closest to those of the aluminium disc. It can therefore be concluded that the MJP AM technology with a selected polymer can be used to verify designed geometries in high-speed machines. The mechanical properties and design of the disc require some modifications, as seen in the estimated results. To improve performance, it would be necessary to optimise the disc in terms of its strength, particularly the strength of the compressor blades. In this study, the disc was tested for the possibility of using MJP technology to verify new and optimised geometries in high-speed turbomachines. The results of the experimental studies confirmed the aims of the paper.

The operating properties of elements manufactured using the AM technology make it possible to extend the experimental verification. Destruction tests performed on the discs helped to determine the maximum speed at which the compressor disc, made of light-cured polymer resin, can operate safely (100,000 rpm). This value was determined based on the stress values that occur under these operating conditions and the speed at which the rotor is destroyed (118,000 rpm).

Nowadays, machines and their components are being optimised in a way that makes it possible to create modelled parts using available conventional manufacturing methods such as machining, CNC milling or casting. The authors of this paper, after analysing the measurement data, propose to use MJP technology as the most accurate AM technology for optimising precision parts as an experimental verification. First of all, it is worth pointing out that this manufacturing method can be somewhat universal in this field, having very many advantages, but also limitations. One of the disadvantages of this method is the limitation of the operation of the manufactured part in the above-mentioned range, while in rotating machines of similar dimensions, it is possible to successfully test the printed model in the range of 0–100,000 rpm. Despite this limitation, it is a very wide range. Another disadvantage is the operating temperature of the polymer used in this technology. According to the manufacturer’s specifications, the VisiJet M3-X material is not capable of operating at a temperature of 88 ${}^{\circ }$C and stresses of around 0.45 MPa. Based on experimental studies, its maximum operating temperature is estimated to be equal to 70 ${}^{\circ }$C when the stresses are around 40 MPa. Apart from these limitations, when it comes to producing prototypical parts for verification tests, it is a method that is much cheaper and faster in terms of creating very complex geometries compared to conventional methods.

**Figure 11:**
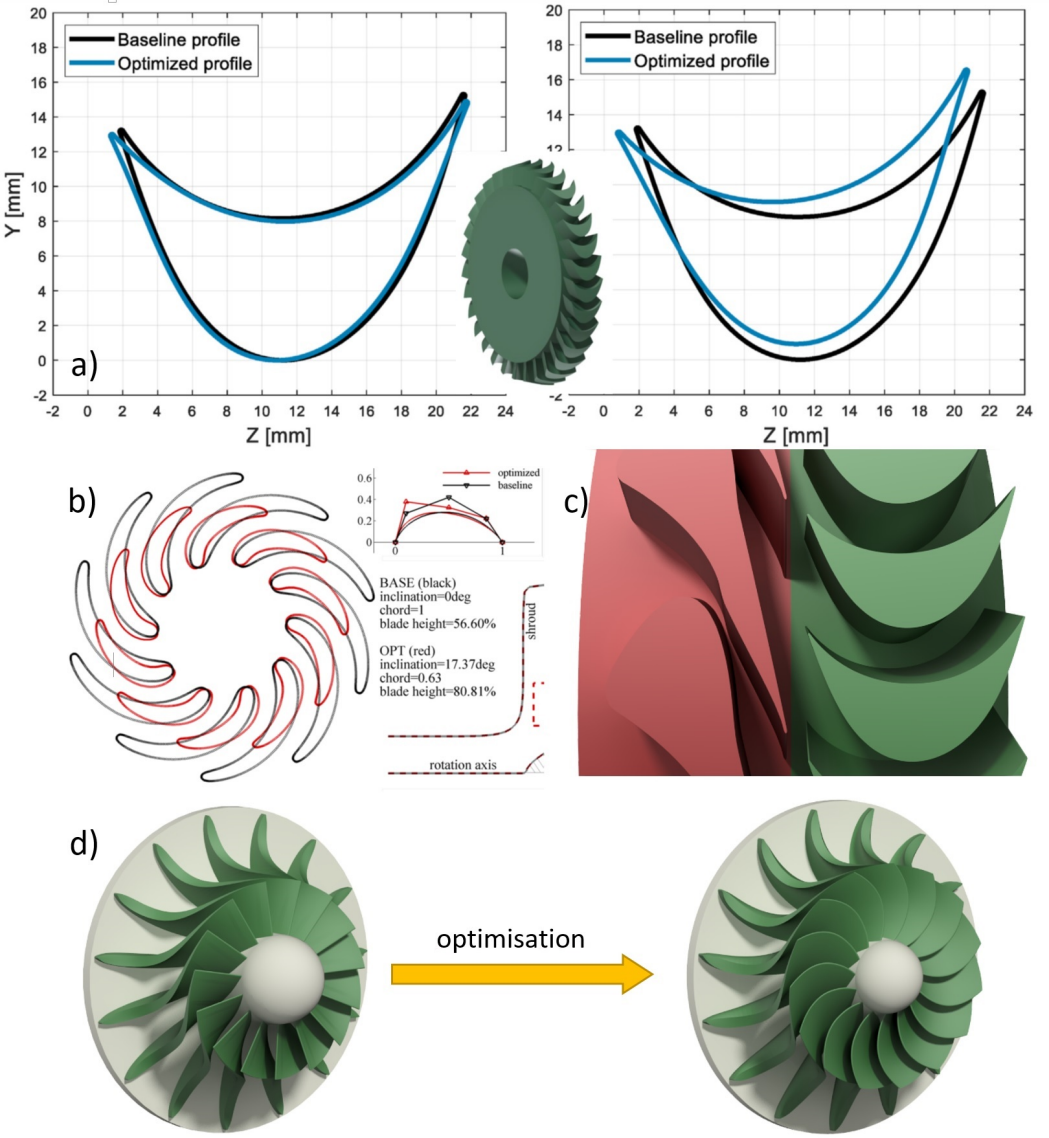
Examples of geometry optimisations carried out on fluid-flow machines: (**a**) optimisation of the geometry of the blades of a gas microturbine [45]; (**b**) optimisation of the rotor of an air-breathing radial outflow turbine [46]; (**c**) optimisation of the guide vanes of an ORC microturbine [47]; (**d**) optimisation of the disc of a radial-axial turbine [37].

Additive manufacturing techniques are currently used to optimise production processes that are based on conventional methods [44], i.e., casting, etc. The method proposed by the authors could be used to produce prototypical parts that would be ready for testing. A result of such an optimisation can be a simple geometry like in [45,46], where the geometry was optimised to improve the flow performance and thus the efficiency of the machine (a, b). In such cases, optimisations can be carried out simultaneously in several variants, taking into account economic aspects such as a cheaper and faster manufacturing process. An example of a very complex geometry is the rotor disc optimisation described in [37,47]. Geometry optimisation based on conventional methods can be very difficult, time-consuming and expensive (c) or even impossible (d). As for the last type of optimisation, this method makes it possible to verify geometries that have been the subject of theoretical considerations until now. Examples of application of the proposed method, collected based on the current state of knowledge, are shown in Figure 11.

The future optimisation of the geometry associated with the current paper will be performed using three variants to carry out an experimental verification. The geometry of the polymer disc of the compressor rotor will be optimised in terms of its strength according to the first variant, and in terms of the flow performance according to the second variant, in order to improve the operating characteristics of the machine. In the last variant, the two previous assumptions will be taken into account for the optimisation, with the emphasis put on improving the operating characteristics obtained and their stability over the entire range of rotational speeds mentioned in the paper.

## 5. Conclusions

The paper presents the results of some preliminary studies conducted to validate the use of the parts of high-speed machines, manufactured using the selected AM method (MJP). For this purpose, a car turbocharger was used in the research. The original compressor disc (made of aluminium), which is the object of research, was scanned and then, using a 3D printer, a disc made of light-cured polymer resin was printed. The polymer disc was tested to verify if its nature of operation reflects that of the aluminium disc. Before initiating the experimental research, numerical analyses were conducted regarding the strength of the printed element and the flow characteristics of the turbocharger compressor used.

The results of experimental research were used to determine the extent of the usefulness of the polymer disc in experimental work associated with the design of rotor discs for fluid-flow machines. The range of proper operation of the car turbocharger was 60,000–80,000 rpm. At a speed of 90,000 rpm, small losses due to the poor performance of the blades were observed. Due to the nature of operation of the compressor of this machine, it was not possible to obtain the operating characteristics for speeds below 50,000 rpm (only after reaching 50,000 rpm could the variations in flow rate and pressure on the compression side be measured). For machines whose rotors have similar dimensions, for example, gas turbines (in the case of a polymer disc, these machines can be supplied, for example, with air), the operating range is very wide and ranges from 0 to 80,000 rpm.

Some discrepancies were observed between the results of the experiment and simulation. The different nature of the curves resulted from not taking into account the value of the blade clearance in the numerical model. The numerical model needs to be improved in future studies. Nevertheless, the inclination angles of the curves decreased with increasing speed during the dynamically stable operation of the turbocharger (at speeds between 60,000 and 80,000 rpm), and the results of the experiment and simulation were similar to some extent.

The destructive tests carried out on the polymer disc made it possible to determine both its maximum and permissible rotational speed. The maximum and permissible speed is 118,000 rpm and 100,000 rpm respectively, taking into account the permissible stresses. An additional advantage of the polymer disc is the fact that after the disc was torn into pieces, the remaining components of the turbocharger remained intact and it was possible to continue operating the machine.

This paper has made it possible to examine whether MJP technology can be used for the experimental verification of numerically optimised models. The results confirmed that this technology is well suited for research in this field. The proposed method can be used to carry out multi-variant optimisations of the geometry of the blades of existing high-speed machines.

Future papers will focus on the optimisation of the geometry of the polymer disc used in terms of strength and performance (flow optimisation), as well as on the verification of the theoretical hypotheses based on the experimental results. The results obtained in this paper will also be used to prepare a physical model of a disc made of VisiJet M3-X material in order to perform necessary analyses.

## Data Availability

Data sharing not applicable.
